# Recognition and function analysis of *MKK6* in immune reaction against *Vibrio Parahaemolyticus* in Crassostrea hongkongensis

**DOI:** 10.1371/journal.pone.0360613

**Published:** 2026-10-15

**Authors:** Jiexiong Zhu, Guohui Shao, Jiayu Chen, Jinji Lin, Qiwen Liang, Feifei Yu, Zhiwen Wang, Baixu Lin, Yongyao Huang, Huiyan Li, Junhui Xu

**Affiliations:** 1 Fishery College of Guangdong Ocean University, Zhanjiang, China; 2 Academician Joint Laboratory of Germplasm Resource Exploitation, Utilization and Health Assessment for Aquatic Animal, Guangdong Ocean University, Zhanjiang, China; 3 Guangdong Provincial Key Laboratory of Aquatic Animal Disease Control and Healthy Culture, College of Fisheries, Guangdong Ocean University, Zhanjiang, China; 4 Fangchenggang Vocational and Technical College, Fangchenggang, China; King Abdulaziz University, SAUDI ARABIA

## Abstract

*MKK6* is a key protein kinase that regulates inflammatory response under pathogen infection by activating MAPK signal pathway. However, the function of *MKK6* remained unclear within oysters. Our research recognized a novel *KK6* gene from *C. hongkongensis*, which is named *ChMKK6*. The cDNA of *ChMKK6* gene was 2,760 bp in length, containing an ORF of 1,032 bp encoding 344 amino acids, with a conserved S-TKc region and a DVD region. As illustrated by amino acid alignment and phylogenetic tree, *ChMKK6* shared the maximal (99.0%) identity showing *MKK6* of *Crassostrea gigas.* As indicated by tissue expression analysis, *ChMKK6* possessed the maximal expression within hepatopancreas, and was markedly increased in all tissues following *Vibrio Parahaemolyticus* (*V. Parahaemolyticus*) challenge. As stated by the qRT-PCR analysis, *ChMKK6* silencing significantly suppressed *IL17* transcription, suggesting its involvement in innate immunity by regulating *IL17*-mediated inflammatory response. Moreover, *ChMKK6* silencing led to the suppression of key immune-related enzyme activities (ACP, AKP, CAT, LZM, and SOD), while raising the MDA content in gill. In addition, the antimicrobial viability of hemolymph supernatant was markedly reduced by *ChMKK6* silencing. These results collectively demonstrate that *ChMKK6* is a key player in immunity defense of *C. hongkongensis* from *V. Parahaemolyticus.*

## 1. Introduction

As an invertebrate, oysters lack the specific immune system like vertebrates and evolve an unique innate immune system to defend against invasion of exogenous microorganisms [1]. Due to continuous expansion of aquaculture scale and the deterioration of aquaculture environments, the oyster has experienced acute infections caused by parasites, bacteria, and viruses, especially gram-negative and facultative anaerobic vibrios [2,3]. *Vibrio parahaemolyticus* (*V. parahaemolyticus*) is widely distributed in estuarine and coastal waters, and is regarded as a primary pathogen in oyster farming [4]. *V. parahaemolyticus* exhibits strong pathogenicity in filter-feeding oysters by adhering to the host's intestinal mucosa, stimulating the release of inflammatory factors, and ultimately inducing an intestinal inflammatory response [5]. This pathogen is commonly associated with mass mortality events in farmed oyster populations [6].

The MKK family members were important regulatory factors, and served as pivotal intermediate activators in the MAPK signaling pathway [7]. These MKK proteins serve as critical intermediaries, linking upstream kinases (e.g., MAPK3) with downstream MAPKs (such as ERK, JNK, and p38) to transmit extracellular signals and regulate essential cell processes, like growth, differentiation, and apoptosis. The MKK family have the identical conserved domains: a serine/threonine protein kinase (S-TKc) region with conserved dual phosphorylation sites (231 Ser and 235 Thr), and a conserved DVD region [8]. MKK6 works as an important signal transduction protein within the p38 MAPK pathway, and could be phosphorylated via upstream MKKs, and activates p38 MAPK by phosphorylation [9]. Recently, *MKK6* have drawn vast attention for their key roles in intrinsic immunity [10,11]. To be specific, Su et and colleagues cloned *MKK6* gene from Orange-spotted grouper (*Epinephelus coioides*), while observing a notable upregulation of its expression in both cephalothorax and spleen following bacterial challenge, with a significant decline after 24 hours [11]. In White shrimp (*Litopenaeus vannamei)*, knockdown of *Lv*MKK*6* suppressed WSSV replication during 24–144 h post infection and reduced VP28 gene expression between 48 and 120 h[10]. The *MKK6* of *Apostichopus Japonicus Selenka* was also characterized and shown to be highly expressed in coelomocytes after *Vibrio splendidus* (a pathogenic vibrio) infection [12].

*Crassostrea hongkongensis* (*C*. *hongkongensis*) works as an economically crucial oyster species, and widely cultivated within the southern China [13]. In recent years, *Vibrio* infections have presented an acute risk to oyster survival and aquaculture. Understanding the immune system of *C. hongkongensis* from *Vibrio* is essential to enhance the oyster`s disease resistance. As illustrated by our prior transcriptomic analysis, *MKK6* transcript was significantly upregulated after *Vibrio parahaemolyticus* (*V. parahaemolyticus*) challenge [14]. Therefore, it is speculated that *MKK6* involved in immune reaction from *V. parahaemolyticus* within *C. hongkongensis*.

Our research characterized a new *MKK6* gene from *C. hongkongensis*. Its expression profile was construed before and after *V. parahaemolyticus* infection. The function of *ChMKK6* in innate immune reaction were validated by RNAi, qRT-PCR, immune enzyme analysis, and antimicrobial activity detection.

## 2. Materials and methods

### 2.1. Oyster rearing, bacterial challenge and experimental sample collection

In Zhanjiang, China, the normal *C. hongkongensis* samples were gathered from a fishery farm. On average, these animals weighed 150.0 g ± 15.0 g, and was 10.0 cm ± 1.0 cm long. 80 samples were segregated stochastically into four groups and separately maintained within four sea water-containing tanks (volume of each tank: 30 L) at constant temperature (24.0 ± 0.5℃) and salinity (20.0 ± 0.5) for one week before experiment.

*V. parahaemolyticus* was cultivated in TCBS medium and suspended in sterilized phosphate buffer saline (PBS) at the ultimate concentration of 1 × 10^9^ CFU/mL. The 100 µL bacterial suspension got an injection into adductor of each individual in bacteria challenge group, and 100 µL of PBS in control group. Nine individuals were repeated in each group. Following a bacterial challenge for 6 h, hemolymph was extracted from the oyster`s pericardial cavity and centrifuged at 3000 rpm for 5 min at 4.0 ℃ to isolate hemocytes from hemolymph supernatant (HS). Blood cells were collected for cDNA amplification, and HSs were used for antibacterial activity analysis. Gill tissues were utilized for malondialdehyde (MDA) content analysis, gene expression, and immune enzyme activity assays. Tissue expression profiling was conducted using hepatopancreas, gills, adductor muscles, mantle, heart, gonads, and blood cells.

### 2.2. RNA extraction and cDNA synthesis

Total RNAs were obtained from hemocytes with the Spin Column Animal Total RNA Purification Kit (Sangon). RNA quality was gauged by electrophoresis on 1.0% agarose gels. Its quantity was gauged through absorbance at 260nm with NanoDrop ND1000 Spectrophotometer. The single strand cDNA underwent synthesis from total RNA with Superscript II polymerase kit (TransGen) and stochastic primers.

### 2.3. The *ChMKK6* cDNA cloning and sequence analysis

As to *MKK6*, the complete cDNA was cloned via SMART RACE cDNA Amplification Kit, complying with the manufacturer’s guidelines. The primers (*ChMKK6*-outer-F and *ChMKK6*-outer-R) were devised according to partial sequences from transcriptome data. Nested-PCR was performed via *ChMKK6*-inner-F and *ChMKK6*-inner-R, aimed at enriching the DNA band. The test-PCR was utilized for checking the validity of related sequences by *ChMKK6*-test-F and *ChMKK6*-test-R. In Table 1, total primers were devised through Primer Premier 5.0.

**Table 1:** Primers in this study.

Gene name	Forward primers / Reverse primers(5′-3′)	Purpose
*ChMKK6*-outer-F	ACACGTCCGAATTATGTTGAACTCCT	3’RACE
*ChMKK6*-outer-R	GTGTCGCACCAAGTCTACGA	5’RACE
UPM	TAATACGACTCACTATAGGGCAAGCAGTGGTATCAACGCAGAGT	RACE universal primer
*ChMKK6*-inner-F	TCATCAAGAGGGGTGAGGAGAAGC	nest-3’RACE
*ChMKK6*-inner-R	GCCCCAGATTTCGGATGGTG	nest-5’RACE
NUP	AAGCAGTGGTATCAACGCAGAGT	Nest-RACE universal primer
*ChMKK6*-test-F	AACAGTCAGGAGCAGAAG	cDNA test
*ChMKK6*-test-R	CCAGTAGCCAACTCAATC	cDNA test
*ChMKK6*-siRNA-F	GCGTAATACGACTCACTATAGGGTCGCTGTTAAGAGAATTCCTATG	RNAi
*ChMKK6*-siRNA-R	GCGTAATACGACTCACTATAGGGAATATCATAGCCTTTTTGTCCAG	RNAi
*β-actin*-F	GTGCTACGTTGCCCTGGACTT	qRT-PCR
*β-actin*-R	TCGCTCGTTGCCAATGGTGAT	qRT-PCR
GFP-siRNA-F	GATCACTAATACGACTCACTATAGGGATGGTGAGCAAGGGCGAGGA	RNAi
GFP-siRNA-R	GATCACTAATACGACTCACTATAGGGTTACTTGTACAGCTCGTCCA	RNAi

In terms of *ChMKK6*, the complete cDNA was construed through the Blast tool in NCBI. The open reading frame (ORF) was located by the ORF finder tool. Protein molecular weight and theoretical isoelectric point (PI) were construed through ExPasy. Transmembrane structural domains and signal peptides were forecasted via TMHMM program and SignalP program. Multiple sequence comparison and phylogenetic tree (PT) construction were conducted via DNAman (Lynnon Biosoft, Canada) and MEGA7. DNAstart was utilized for figuring out the identity of *MKK6* sequences from different species.

### 2.4. Quantitative real-time PCR (qRT-PCR) analysis

The qRT-PCR was carried out by the LightCycler 96 Instrument (Roche Diagnostics, Switzerland) according to the manufacturer’s protocol of PerfecterStart^®^ Green qPCR SuperMix. The reaction was performed within 20.0 µL reaction mixture, comprising 0.5 µL cDNA, 0.4 µL of each primers, 10.0 µL SYBR Green Master Mix and 8.7 µL Nuclease-free Water (TransGen, Beijing, China). PCR parameters reached 95.0 ℃ for 5 min, before 45 cycles of 95.0 ℃ for 10 s, 59.0 ℃ for 15 s and 72.0 ℃ for 15 s. Table 1 displayed the related primers. The β-actin was utilized as an internal reference. Each reaction was executed thrice. Relative gen expression was computed by the 2^-ΔΔCt^ method.

### 2.5. RNA interference experiment

*ChMKK6*-siRNA1 and *ChMKK6*-siRNA2 underwent synthesis to specifically silence STKc and DVD structural domain of *ChMKK6*, respectively. *GFP*-siRNA was obtained by chemical synthesis from pEGFP-N3 plasmid as a negative control (NC) (primers as Table 1). The siRNA was synthesized through T7 High Efficiency Transcription Kit (TransGen, China) and underwent purification using EasyPure RNA Purification Kit (TransGen, China).

Each oyster underwent an intramuscular injection of 50.0 µL of 1.0 μg/µL siRNA and was reinjected with the identical amount on Day 3, aimed at improving the silencing influence. On Day 5, each oyster underwent an injection of 100.0 µL suspension at a concentration of 1 × 10^9^ CFU/mL, with the intention of activating an immune reaction. Each experimental group included five samples. After 6 h bacterial challenging, gills were gathered to extract RNAs, to determine non-specific immune enzyme activity, and to analyze MDA. At 4.0 ℃, hemolymph was withdrawn and underwent centrifugation at 3000 rpm for 5 min to segregate the HS and hemocytes for antimicrobial activity assay.

### 2.6. Non-specific immune enzyme activity assay

Activities of superoxide dismutase (SOD), catalase (CAT), acid phosphatase (ACP), alkaline phosphatase (AKP) and lysozyme (LZM) were determined via the SOD assay kit, CAT Enzyme Activity Kit, ACP Assay Kit, AKP Assay Kit and LZM Activity Kit (Jiancheng, China) according to prior methods [15,16]. The absorbance was measured at 550 nm (SOD), 450 nm (CAT), 520 nm (ACP and AKP), and 530 nm (LZM), respectively. MDA content was gauged via the MDA kit (Jiancheng, China) at an absorbance of 532 nm. The MDA content was expressed as nmol each mg protein.

### 2.7. Antibacterial viability assay of HS

In terms of HS, the antimicrobial viability was examined according to previous methods [17]. The protein concentration of HS from NC, siRNA1 and siRNA2 groups were modulated to 1.5 mg/ml through Nanodrop ultra-micro spectrophotometer (Thermo scientific, USA). 50 μl sterile HS was blended with 50 μl *Vibrio parahaemolyticus* at a density of 1 × 10^4^ CFU/ml, and cultivated within 100 µl 2116E liquid substrate with shaking for 2 h at 37.0 ℃. Within the blank group, 50 μl PBS was utilized for replacing HS. After shaking, 100 μl mixture was uniformly distributed in TCBS solid medium (Land Bridge, China) and nurtured for 12 h at 37.0 ℃. The quantity of the observed colonies was figured out.

### 2.8. Statistical analysis

Significance analysis between samples was executed via SPSS 25.0 (IBM, USA). *P* < 0.05 reached remarkable disparity, with *P* < 0.01 reaching high remarkable disparity. Statistical plots of results were drawn via Graphpad 8.0.2 (Dotmatics, USA).

## 3. Results

### 3.1. Cloning and sequence analysis on *MKK6* cDNA in *C.hongkongensis*

Through RACE-PCR, the full-length cDNA of *MKK6* was extracted from *C. hongkongensis* and called as *ChMKK6* (GenBank accession NO.: OP292967). In Fig 1, the full-length nucleotide sequence of *ChMKK6* reached 2760 bp in length, like an open reading frame of 1032 bp (244–1276), a 5^′^ UTR of 241 bp and a 3^′^ UTR of 1484 bp with a typical polyadenylation signal sequence (aataaa) and a 32 bp poly (a) tail. The putative amino acid sequence meant 344 amino acids long. No signal peptides and transmembrane domain were discovered within putative *Ch*MKK6 protein. The deduced molecular mass of *Ch*MKK6 protein reached 101.8 kDa showing a PI of 6.49.

**Fig 1:**
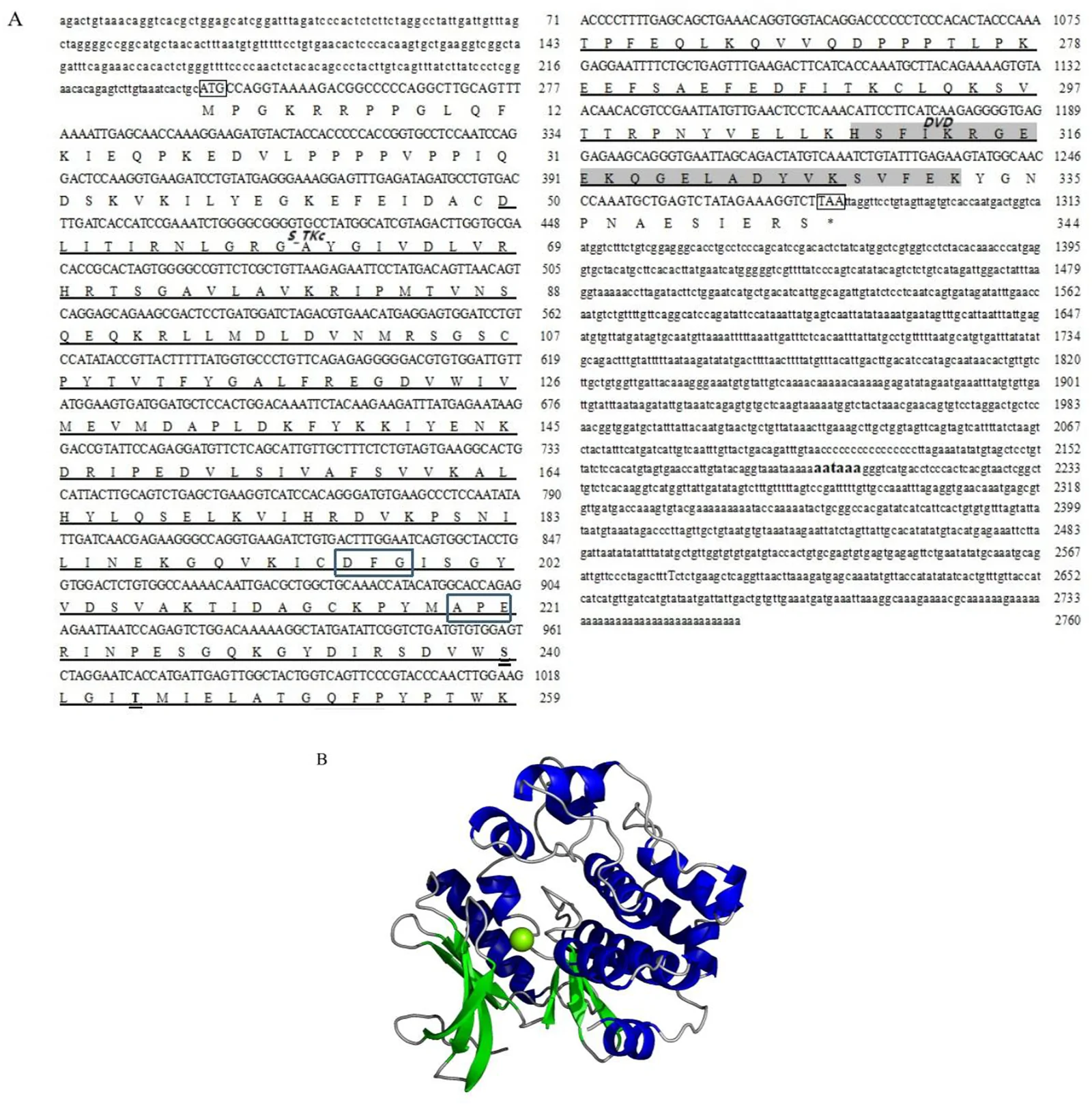
Sequences and tertiary structure of *Ch*MKK6. (A). Nucleotide and deduced amino acid sequences of *ChMKK6.* Uppercase letters meant the ORF sequence. Lowercase letters meant 5 ‘UTR and 3’ UTR sequences. The start codon (ATG) and the stop codon (TAA) were black boxed. The hypothetical S_TKc structural domain was marked with a horizontal line and the DVD structural domain was marked with a gray box. DFG and APE were marked with a blue box. The conserved dual phosphorylation sites (240 Ser and 244 Thr) were shown by double underline. Hypothetical polyadenylation signals (aataaa) were marked in bold. * Indicated that no amino acid was coded. (B) Tertiary structure of *Ch*MKK6 protein.

The putative *Ch*MKK6 protein was found to contain a conserved serine/threonine protein kinase catalytic structural domain (S_TKc) of 278 amino acids and a docking structural domain (DVD) of 24 amino acids (Fig 1A). The S_TKc domain was known to be responsible for protein phosphorylation and ATP binding, while the DVD domain facilitates for MKK activation via upstream MKKKs in the pathological reaction. The S_TKc included DFG (Asp-Phe-Gly) and APE (Ala-Pro-Glu) motifs which regulated ATP-binding interactions. The conserved dual phosphorylation sites (Ser and Thr) were located at residue 240 and 244 of this protein.

### 3.2. Multiple sequence alignment and phylogenetic analysis

Amino acid sequence alignments were executed for MKK6 from *C. hongkongensis* and others. The *Ch*MKK6 shared the maximal (99.0%) identity showing MKK6 of *C. gigas*, 99.0% identity with that of *C. virginica*, 94.0% with *Mizuhopecten yessoensis*, and 81.0% with *Haliotis rufescens*. The lowest identity (42.1%) was observed between MKK6 from *Aplysia californica* and *C. hongkongensis*. As noted by amino acid sequence comparison, the conserved S_TKc domain and DVD domains emerged in MKK6 from all molluscs examined, including *C. hongkongensis*, *C. gigas*, *C. virginica*, *Mizuhopecten yessoensis*, *Mytilus coruscus*, *Mytilus galloprovincialis*, *Haliotis rufescens, Pomacea canaliculate* and *Aplysia californica* (Fig 2A).The phylogenetic tree analysis was executed to illustrate the evolutionary relationship of *MKK6* genes from discrepant species (Fig 2B). The *Ch*MKK6 proteins from molluscs referred above were grouped into a large clade. This indicated that they possessed a common ancestor. The MKK6 amino acid sequence of vertebrates were clustered into a close branch, including *Homo sapiens, Epinephelus coioides* and *Xenopus laevis,* exhibiting low homology with *Ch*MKK6. The MKK6 of *Drosophila navojoa* exhibited farther distance to that of molluscs and vertebrates.

**Fig 2:**
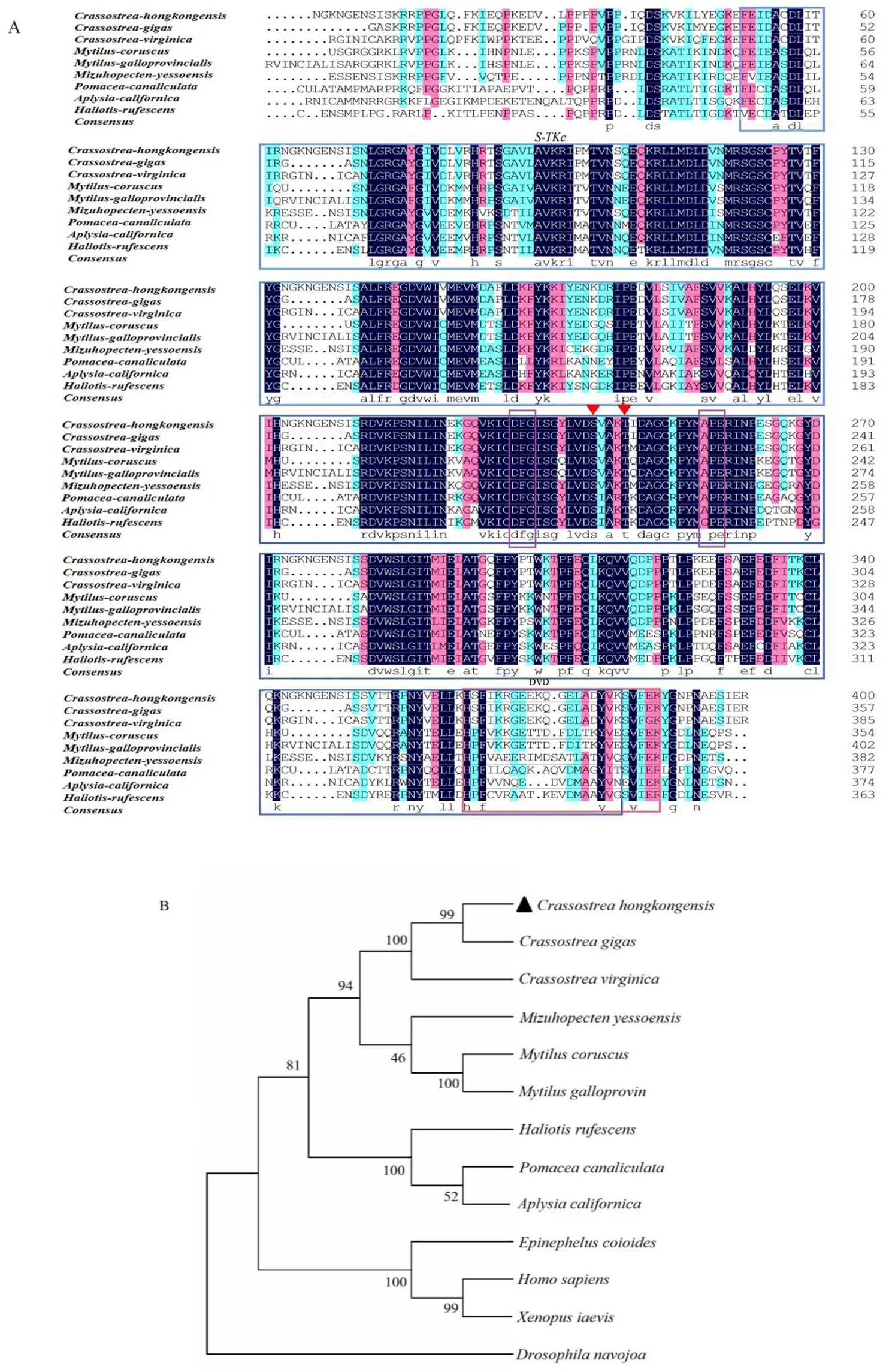
Sequence alignment and phylogenetic tree analysis. (A). Multiple sequence comparison of *MKK6* in *C. hongkongensis* and other molluscs. The same residues were indicated in black, highly conserved residues in red, and conserved residues in blue. S_TKc and DVD structural domains were boxed in blue and red, separately. The DFG and APE motifs were boxed in purple. The dual phosphorylation sites (240 Ser and 244 Thr) were shown by red arrow. (B). PT of MKK6. The PT was formulated by the adjacent joining method. Numbers on nodes denoted bootstrap values gauged from 1000 repeated bootstrap analyses. The species names and Gen Bank accession numbers were listed below: *Crassostrea hongkongensis* (OP292967), *Crassostrea gigas* (XP_011423486.2), *Crassostrea virginica* (XP_022319507.1), *Mizuhopecten yessoensis* (XP_021376386.1), *Mytilus coruscus* (CAC5366754.1), *Mytilus galloprovincialis* (AJQ21545.1), *Aplysia californica* (XP_012936929.1), *Pomacea canaliculata* (XP_025096480.1), *Homo* *sapiens* (NP_001317379.1), *Epinephelus coioides* (QBQ65829.1), *Xenopus laevis* (NP_001079947.1), *Drosophila navojoa* (XP_017964700.1) and *Haliotis rufencens* (NC_002431).

### 3.3. ChMKK6 mRNA expression profile before and after *V. Parahaemolyticus* challenging

Expression levels of *ChMKK6* among discrepant tissues before and after challenge were determined by qRT-PCR with β-actin as a reference. In Fig 3, prior to *V. Parahaemolyticus* challenge, *ChMKK6* exhibited the maximal expression level within hepatopancreas, higher expression in gill, heart, adductor muscle, gonad and mantle, and the lowest expression within hemocytes (*P* < 0.05). Following *V. Parahaemolyticus* challenging, *ChMKK6* transcripts were prominently upregulated by 27.3% in hepatopancreas (*P* < 0.01), 42.8% in gill (*P* < 0.01), 56.9% in heart (*P* < 0.01), 51.2% in adductor (*P* < 0.01), 20.7% in gonad (*P* < 0.05) and 28.7% in hemocytes (*P* < 0.05). No remarkable change distributed in mantle.

**Fig 3:**
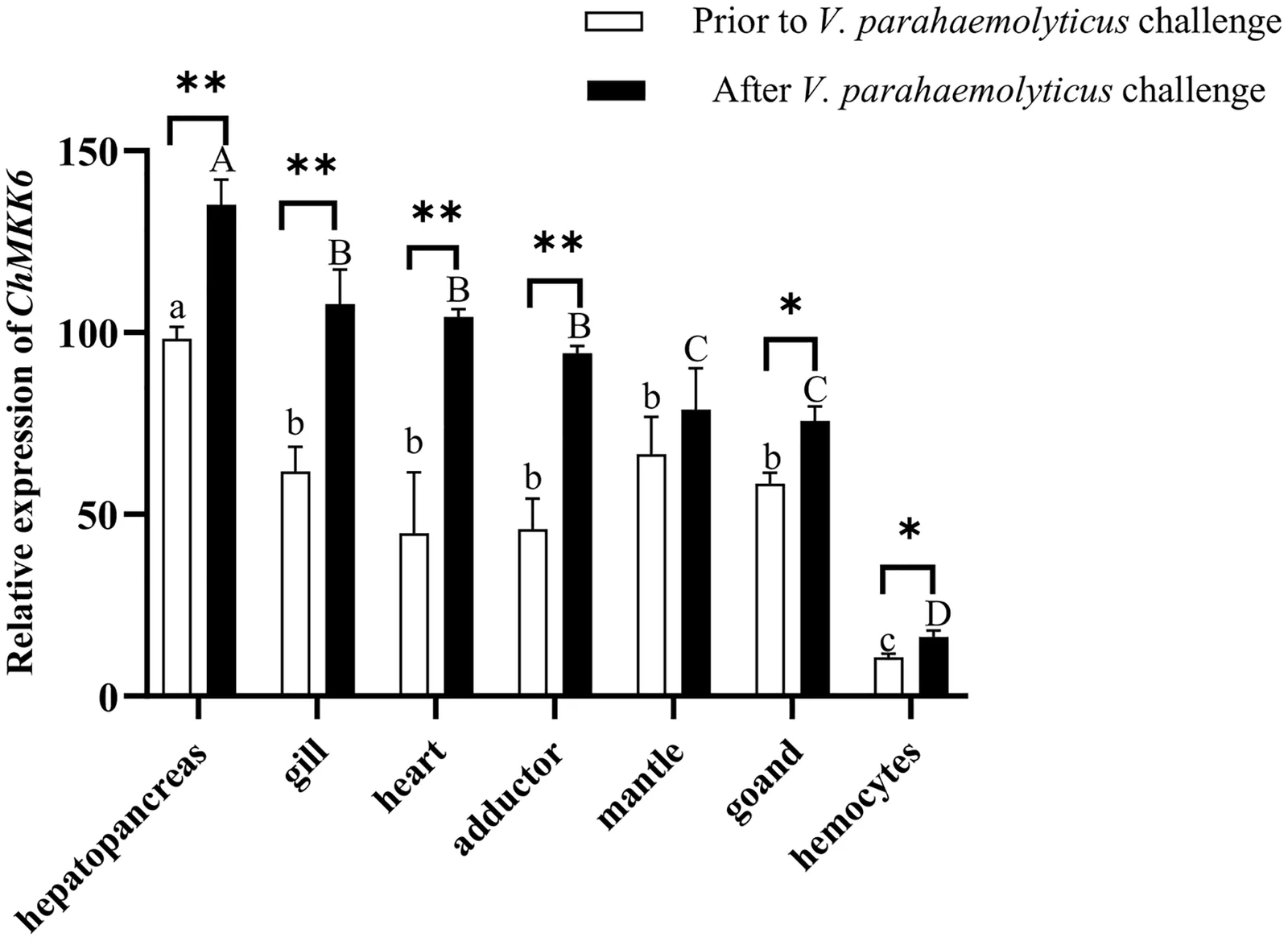
Relative MKK6 expression among tissues prior and subsequent to V. Parahaemolyticus challenge. The β-actin of C. hongkongensis was utilized as a reference. Discrepant lowercase letters represented remarkable disparity (P  <  0.05) among tissues prior to V. Parahaemolyticus challenge, and different capital letters meant significant differences (P  <  0.05) among tissues after Vibrio challenge. The difference before and after Vibrio challenge was indicated using * (P  <  0.05) or ** (P  <  0.01) (N  =  5)).

### 3.4. *ChMKK6* silencing inhibited the transcription of *IL17*

The transcriptional levels of immune genes were detected after RNAi and Vibrio challenge. Relative to the NC group, the *ChMKK6* expression was markedly reduced by 42.2% and 37.4% (siRNA1, siRNA2, *P* < 0.05) (Fig 4), indicating that *ChMKK6* was successfully silenced. *IL17,* an early inducer of inflammatory responses, was significantly reduced by 45.5% (siRNA1, *P* < 0.01) and 14.4% (siRNA2, *P* < 0.05) by *ChMKK6*, respectively (Fig 4). No significant change was found in *ChTLR4*, *ChMYD88*, *ChIRAK4*, *ChTRAF6, ChHSP90* and *ChCOX2* transcripts. Findings showed that *ChMKK6* might engage in immune reaction against *V. Parahaemolyticus* by *ChIL17*-mediated inflammatory responses*.*

**Fig 4:**
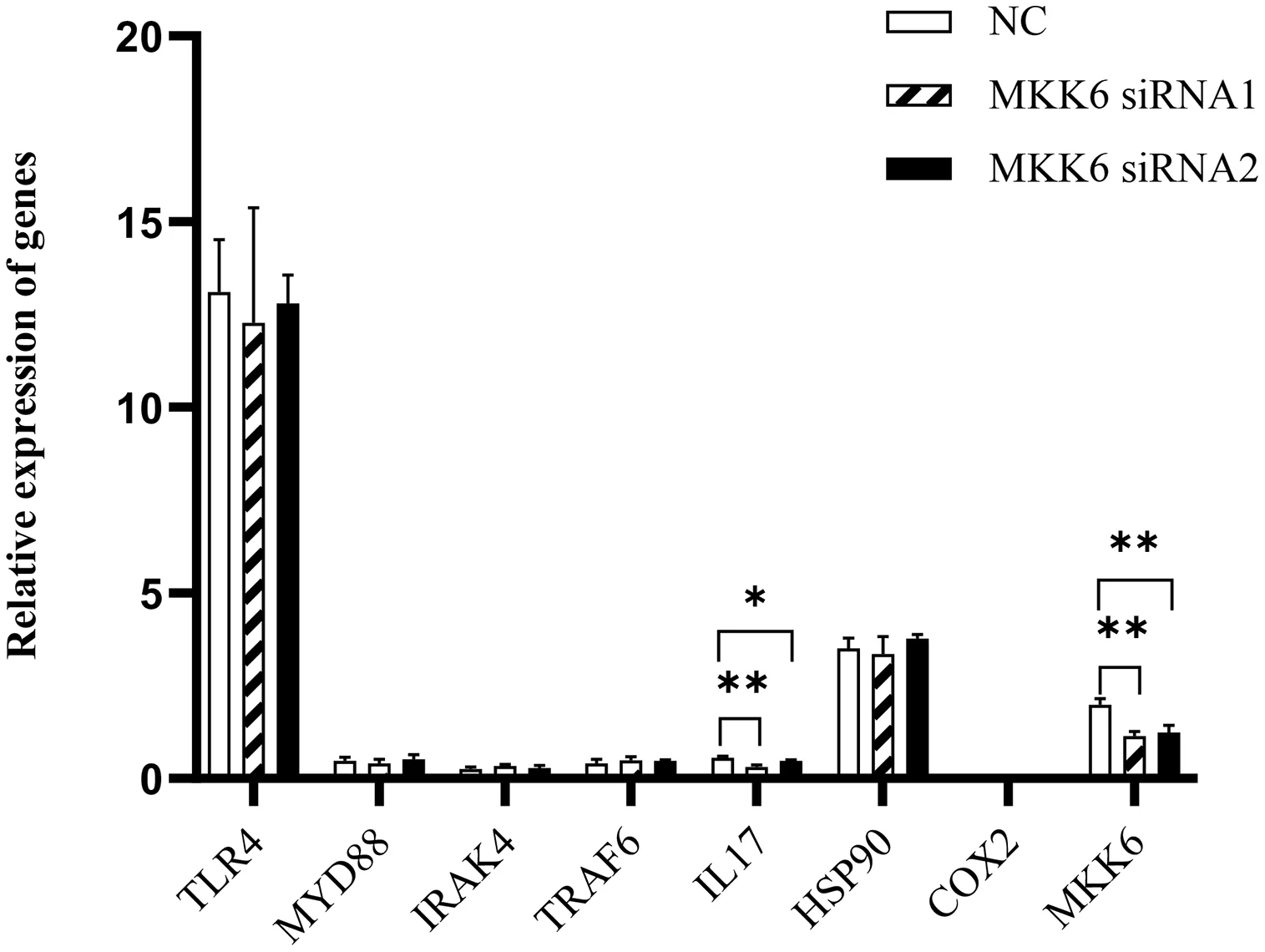
Expression analysis on immune-associated genes in *C. hongkongensis.* The *β-actin* of was utilized as a reference. Each bar was an average of 5 specimens * (*P* < 0.05) and ** (*P* < 0.01).

### 3.5. Immune Enzyme activities were inhibited following *ChMKK6* silencing

To investigate the role of *ChMKK6* in immune reaction from *V. Parahaemolyticus,* immune enzymatic activities and MDA content were measured. Compared with NC group, *ChMKK6* silencing resulted in a significant reduce in SOD viability by 42.7% and by 28.8% (siRNA1, siRNA2, *P* < 0.05) (Fig 5A). MDA content was consistently raised by 129.7% and 151.9% (siRNA1, siRNA2, *P* < 0.01) (Fig 5B). ACP activity was significantly suppressed by 39.1% and 49.6% (siRNA1, siRNA2, *P* < 0.01) (Fig 5C). Similarly, AKP activity reduced by 41.7% and 36.8% (siRNA1, siRNA2, *P* < 0.05) (Fig 5D), CAT activity decreased by 46.5% and 50.9% (siRNA1, siRNA2, *P* < 0.01) (Fig 5E), and LZM activity declined by 45.8% and 30.1% (siRNA1, siRNA2, *P* < 0.05) (Fig. 5F). This results indicate that *ChMKK6* enhances immune enzymatic activities and promotes immune activation in *C. hongkongensis*.

**Fig 5:**
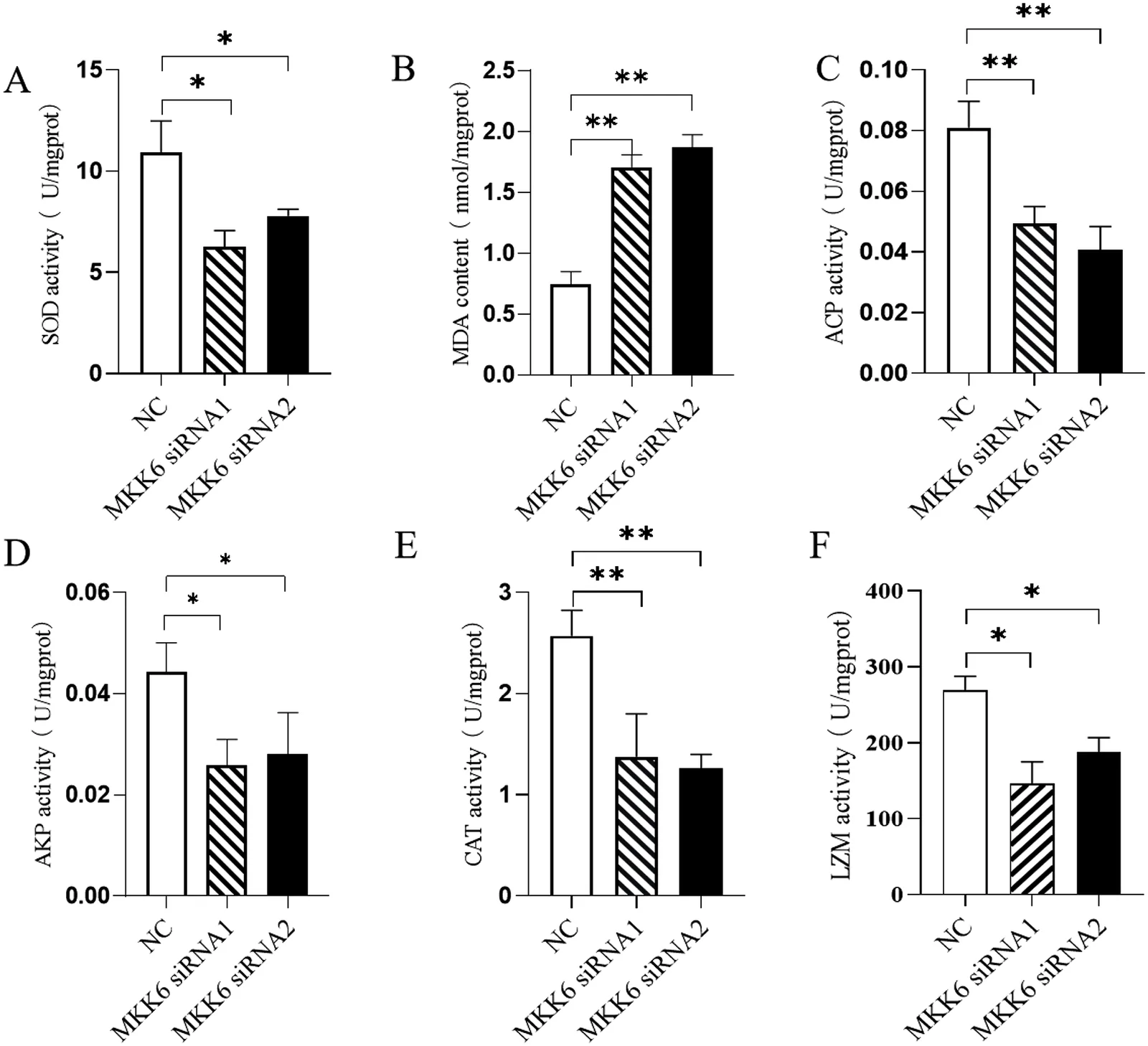
The non-specific immune enzymatic viability following *ChMKK6* silencing in C. hongkongnsis. (A). SOD activity (B). MDA content (C). ACP activity (D). AKP activity (E). CAT activity (F). LZM activity. Error bars represented standard deviation, * (*P* < 0.05) and * * (*P* < 0.01) (N = 3).

### 3.6. *ChMKK6* silencing suppressed the antibacterial viability of HS in *C. hongkongensis*

The antibacterial viability of HS was evaluated based on its inhibitory effect on *V. Parahaemolyticus*. As showed in Fig 6, relative to the blank group, the number of colonies in negative control (NC) was significantly reduced, indicating a strong antibacterial function of the hemolymph supernatant from *C. hongkongensis*. Notably, in the siRNA groups, the colony count increased significantly by 75.0% and 80.8% (siRNA1, siRNA2, *P* < 0.05) relative to the NC group. These results suggest that *Ch*MKK*6* silencing substantially impairs the antibacterial viability within experimental oysters.

**Fig 6:**
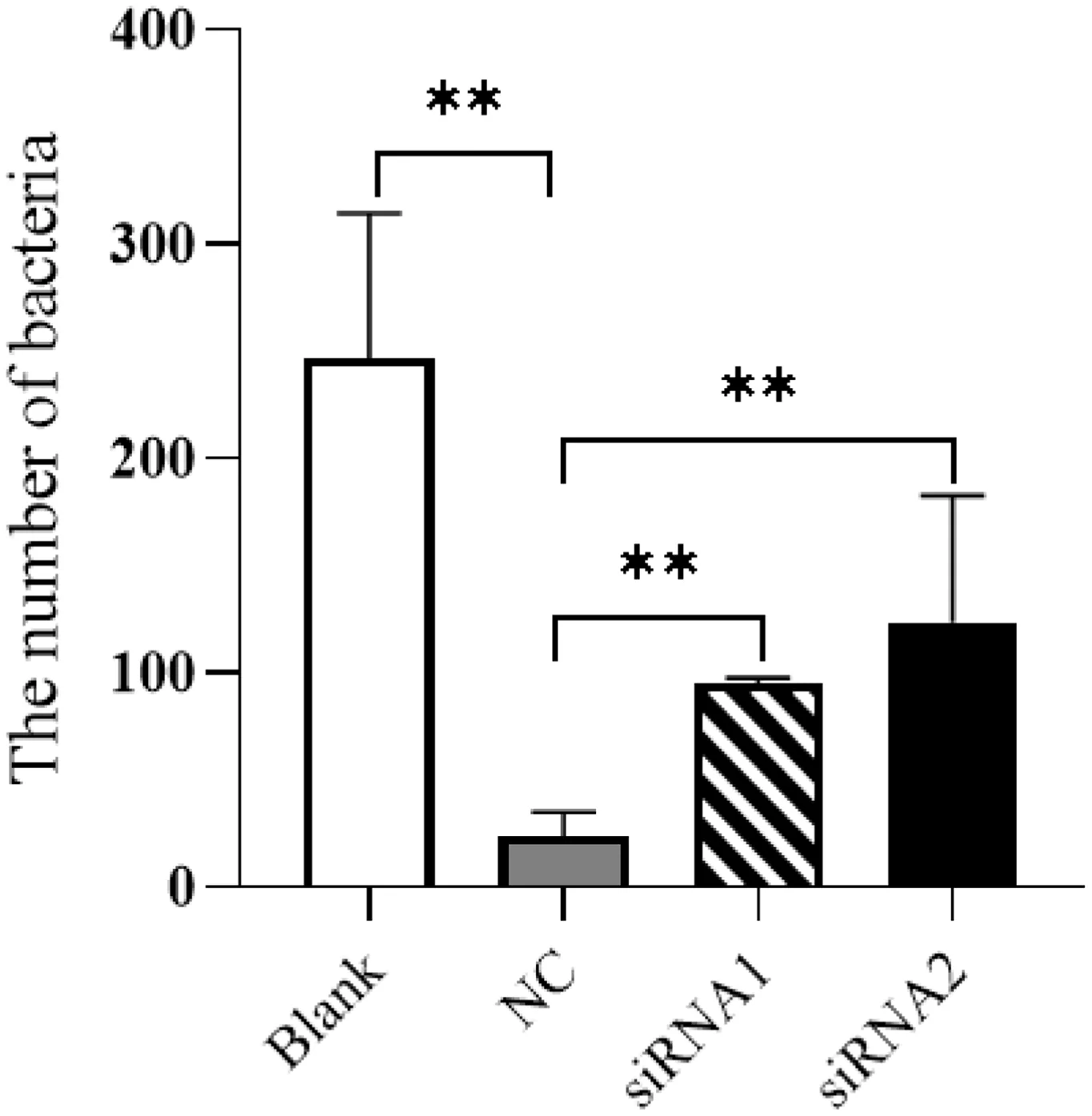
Antibacterial activities of hemolymph supernatant from blank group, NC group, *ChMKK6*-siRNA1 group and *ChMKK6*-siRNA2 group. Data were described by mean ± SD (N = 3). * (*P* < 0.05) and ** (*P* < 0.01).

## 4. Discussion

Aquaculture has evolved into the most efficacious food-producing sector. It supplies a third of all animal protein foods in China, and contributes about 60.0% of global aquaculture output [18]. *C. hongkongensis* is an important farmed shellfish species in southern China, with high nutritional value and economic status. However, in recent years, frequent outbreaks of Vibrio-related mortality have been reported [11,19]. Enhancing disease resistance in oysters is therefore critical for sustainable aquaculture. The research in immune system of *C. hongkongensis* will assist in discovering disease-resistance strategy and enhance the disease-resistance capability of oysters.

In this research, the *ChMKK6* was cloned and structurally analyzed. Similar to other *MKK6*, the *ChMKK6* contained a conserved S_TKc region and a DVD domain [10,20]. The S_TKc domain constitutes the core catalytic region of serine/threonine kinases (STKs), responsible for catalyzing the delivery of the phosphate group from ATP to substrate proteins. This domain generally comprises approximately 250–300 amino acids, featuring a N-terminal rich in glycine for ATP binding and phosphate transfer, and a C-terminal region for substrate binding [21]. Its core function relies on a highly conserved aspartic acid residue, which is key to the enzyme's catalytic activity. The DVD domain works as an about 20-amino acid region situated at the C-terminus of MKK6. Mutations in this domain inhibited the activation of MKK6 phosphorylation by upstream MAPKKKs (Takekawa et al., 2005), thereby disrupting MAPK pathway signaling and the downstream target gene expression [22]. The high conservation in structure implied the stability of function. It was speculated that *ChMKK6* might be a key player in the intrinsic immune of oysters.

As exhibited by tissue expression profile, *ChMKK6* was highly expressed within immune tissues, particularly in hepatopancreas. Following challenge with *V. Parahaemolyticus*, the *ChMKK6* transcripts were significantly upregulated in total explored tissues, and the maximal expression level still emerged within hepatopancreas. The hepatopancreas primarily contributes to immunity via its phagocytic cells, which engulf and degrade pathogens intracellularly, thereby participating in the host's immune defense. Additionally, as a digestive organ, it secretes enzymes such as proteases and amylases to facilitate nutrient breakdown, potentially modulating the intestinal microenvironment to help maintain immune homeostasis. The high expression of *ChMKK6* in immune tissues suggested that *ChMKK6* might be involved in innate immunity against Vibrio in oysters.

*MKK6* was an important signal transduction protein, while engaging in host defense against pathogene infections [23]. In *Epinephelus coioides*, the *EcMKK6* transcription level was prominently increased through *Vibrio alginolyticus* infection, which induced severe inflammation [11]. Similarly, it was shown that in Pacific white shrimp regulated the transcription of antimicrobial peptides to LPS, *Vibrio parahaemolyticus* and Staphylococcus aureus challenge [10]. To investigate the immune function of *ChMKK6* in oyster during *V. Parahaemolyticus* infection, two siRNA sequences were designed to specially silence the S-TKc and DVD domain. Subsequent changes in the expressions of immune genes were analyzed after *ChMKK6* interference. Interleukin-17 (IL17) is a key pro-inflammatory cytokine central to innate immunity, particularly in defense against extracellular pathogens [11]. It activates NF-κB and MAPK pathways to induce antimicrobial peptides and recruit neutrophils [24]. Similarly, the research in Triangle sail mussel (*Hyriopsis cumingii*) found that IL-17 mRNA was upregulated in hepatopancreas and gills following mantle tissue allograft, suggesting their function in modulating inflammatory responses and enhancing pathogen clearance [25]. In this research, *IL17* was significantly inhibited by ChMKK6 silence, indicating that *ChMKK6* involved in innate immune through *IL17*-mediated inflammatory response.

As known, in TLR4-MyD88-MAPK pathway, upon TLR4 activation, the adapter protein MyD88 recruits IRAK4, which phosphorylates and activates IRAK1 to subsequently engage TRAF6, thereby initiating MAPK and NF-κB pathways to drive inflammatory responses [26]. In this research, the transcriptional levels of TLR4, MyD88, IRAK4, and TRAF6 remained unchanged following *ChMKK6* silencing, indicating that *ChMKK6* does not modulate its upstream genes within the TLR4-MyD88 pathway. This suggests an absence of feedback regulation in the TLR4-MyD88-MAPK signaling cascade in oysters. Meanwhile, COX‑2 (Cyclooxygenase‑2) is an inducible enzyme that catalyzes the conversion of arachidonic acid into prostaglandin H2 (PGH2), which are key lipid mediators of inflammation, pain and fever [27]. No significant change was found in COX2 transcripts after *ChMKK6* silence, indicating that the response of *ChMKK6* to *V. parahaemolyticus* is independent of COX2 regulation.

The immune role of *ChMKK6* was deeply validated via immune enzymes activity analysis. As a filter-feeding animal, shellfish are highly susceptible to invasion by pathogenic bacteria and viruses [26]. Once infected, the host cells generate and accumulate reactive oxygen species (ROS), like singlet oxygen, hydrogen peroxide, and superoxide anion [28]. ROS is capable of activating diverse transcription factors, like STAT, HIF-1α, NF-κB, and AP-1, thereby promoting inflammatory responses and cellular transformation [29]. However, at high concentration, ROS can induce immune dysfunction and induce oxidative injury to cell lipids, nucleic acids, and proteins. To sustain the ROS balance and preserve normal cell function, key antioxidant enzymes, such as SOD and CAT, were upregulated. MDA works as a steady byproduct of lipid peroxidation, as one indirect marker of ROS levels, since it accumulates in cells following oxidative damage [30]. Due to its stability and detectability, MDA is widely used to represent the ROS level [31]. Our data showed that the MDA content were remarkably raised. The viability of SOD and CAT were prominently reduced, relative to control. This indicated that *ChMKK6* silence inhibited the antioxidant capacity of oyster and enhanced the damage degree of organism. The phosphatases (ACP and AKP) are known to support phagocytosis by supplying phosphate groups within intracellular lysosomes during pathogen invasion [32]. In this study, AKP, ACP and LZM activities were all significantly reduced by *ChMKK6* silence, indicating that loss of *ChMKK6* function impairs the organism's capacity to phagocytose and degrade pathogens.

Antibacterial activity of hemolymph supernatant is a well-established functional assay to demonstrate the immune role of specific genes in shellfish. A significant decline in antibacterial capability of hemolymph supernatant can provide a direct evidence for the gene's immune function. This approach has been widely used in bivalve research, such as in *Ostrea edulis* [33*]*, *Crassostrea gigas* [33*]* and *Pteria penguin* [34]. In this report, the hemolymph supernatant of *C. hongkongensis* showed distinct antibacterial activity after *Vibrio* infection, while *ChMKK6* silence significantly suppressed the antibacterial viability of HS. The data of antibacterial viability assessment corresponded to the immune enzyme activity analysis and immune gene expression analysis, jointly proving the crucial effect of *ChMKK6* in immunoreaction from *V. Parahaemolyticus* in *C. hongkongensis.*

## 5. Conclusion

To sum up, a new *MKK6* gene, named *ChMKK6,* was identified and emerged in *C. hongkongensis.* The *ChMKK6* contains a conserved S_TKc domain and a DVD domain, and shares the highest sequence identity with *MKK6* from *C. gigas*. *ChMKK6* was highly expressed in immune tissues*,* especially in hepatopancreas, and was further upregulated following challenge with *V. parahaemolyticus*. The marked reduction in *ChIL17* transcript suggests that *ChMKK6* participated in immune reaction via modulating inflammatory reaction. Furthermore, the immune enzyme viability and antibacterial viability results further confirmed that *ChMKK6* is a pivotal player in intrinsic immune defense of *C. hongkongensis* from *V. parahaemolyticus.*

## Abbreviations

ACP: acid chosphatase
APE: Ala-Pro-Glu
AP-1: activator protein 1
AKP: alkaline phosphatase
ATP: adenosine triphosphate
CAT: catalase
CFU: colony-forming unit
COX2: cyclooxygenase-2
DFG: Asp-Phe-Gly
DVD: double von willebrand factor type D
ERK: extracellular signal-regulated kinase
HIF-1α: hypoxia-inducible factor 1-alpha
HSP90: heat shock protein 90
IL17: interleukin-17
IRAK4: interleukin-1 receptor-associated kinase 4
JNK: c-Jun N-terminal kinase
LPS: lipopolysaccharide
LZM: lysozyme
MAPK: mitogen-activated protein kinase
MDA: malonaldehyde
MKK6: MAP kinase kinase 6
MKKK/MAPKKK: MAP kinase kinase kinase
MYD88: myeloid differentiation primary response 88
NC: negative control
NF-κB: nuclear factor Kappa-light-chain-enhancer of Activated B cells
ORF: open reading frame
p38: p38 MAPK
PBS: phosphate buffer saline
PGH2: prostaglandin H2
PI: isoelectric point
qRT-PCR: quantitative reverse transcription polymerase chain reaction
ROS: reactive oxygen species
siRNA: small interfering RNAs
SOD: superoxide dismutase
STAT: signal transducer and activator of transcription
S-TKc: serine/threonine tyrosine kinase catalytic
STKs: serine/threonine kinases
TCBS: thiosulfate-citrate-bile salts-sucrose agar
UTR: untranslated region
VP28: viral protein 28
WSSV: white spot syndrome virus
RNAi: RNA interference
TLR4: toll-like receptor 4
TRAF6: TNF receptor-associated factor 6
